# Diagnosis of Thyroid Nodules Based on Image Enhancement and Deep Neural Networks

**DOI:** 10.1155/2022/5582029

**Published:** 2022-02-15

**Authors:** Xuesi Ma, Lina Zhang

**Affiliations:** ^1^School of Mathematics and Information Science, Henan Polytechnic University, Jiaozuo 454000, Henan, China; ^2^School of Computer Science and Technology, Henan Polytechnic University, Jiaozuo 454000, Henan, China

## Abstract

The diagnosis of thyroid nodules at an early stage is a challenging task. Manual diagnosis of thyroid nodules is labor-intensive and time-consuming. Meanwhile, due to the difference of instruments and technical personnel, the original thyroid nodule ultrasound images collected are very different. In order to make better use of ultrasound image information of thyroid nodules, some image processing methods are indispensable. In this paper, we developed a method for automatic thyroid nodule classification based on image enhancement and deep neural networks. The selected image enhancement method is histogram equalization, and the neural networks have four-layer network nodes in our experiments. The dataset in this paper consists of thyroid nodule images of 508 patients. The data are divided into 80% training and 20% validation sets. A comparison result demonstrates that our method can achieve a better performance than other normal machine learning methods. The experimental results show that our method has achieved 0.901961 accuracy, 0.894737 precision, 1 recall, and 0.944444 F1-score. At the same time, we also considered the influence of network structure, activation function of network nodes, number of training iterations, and other factors on the classification results. The experimental results show that the optimal network structure is 2500-40-2-1, the optimal activation function is logistic function, and the best number of training iterations is 500.

## 1. Introduction

The incidence of thyroid cancer has been increasing the fastest among all solid malignant tumors. The relatively high incidence that continues to grow makes thyroid cancer one of the most common endocrine malignancies worldwide, currently listed as seventh most common cancer in women and fifteenth most common cancer in men [1]. The research of thyroid cancer has become a topic of widespread concern in medical community and society [2]. The diagnosis of thyroid cancer is a challenging task. Most of thyroid cancers are shown as thyroid nodules, which are frequently detected incidentally during the diagnostic imaging of the neck [3–5]. High-resolution ultrasound is the gold standard test for the identification of thyroid nodules. Thyroid disease is generally diagnosed by visual inspection of ultrasound images. Thyroid nodule imaging improves on diagnosis of thyroid diseases by providing ultrasound images. Based on thyroid nodules ultrasonographic characteristics such as internal composition, echogenicity, calcification, margins, and size, radiologists can report on the risk of thyroid nodules being malignant using a standardized scoring system [6]. When using high-resolution ultrasound, the prevalence of thyroid nodules is as high as 19–68% in a randomly selected population. Since most of the nodules are benign and the percentage of malignant nodules is relatively low (7–15%), it is very important to distinguish benign and malignant thyroid nodules [7].

Most of thyroid nodules are heterogeneous with various internal components, which confuse many radiologists and physicians with their various echo patterns in thyroid nodules ultrasonography. In order to improve the diagnosis rate and reduce the loss caused by misdiagnosis, computer-aided diagnosis (CAD) has been developed to help doctors discriminate nodules from benign or malignancy. In 2007, Savelonas et al. focused on the directional patterns of the image they extracted using radon transformation and proposed a computer-aided diagnosis system based on support vector machine on the data of 66 patients, achieving maximal classification accuracy of 0.89 [8]. In 2011, Ding et al. studied the classification of thyroid nodules using the support vector machine classifier and 125 thyroid nodules consisting of 56 malignant and 69 benign patients [9]. Then, in 2019, Prochazka et al. used 40 thyroid nodules (20 malignant and 20 benign) to extract several features, such as histogram parameters, fractal dimension, and mean brightness value. The authors used random forests and support vector machine to differentiate nodules into malignant and benign classes based on these features [10]. In 2020, Harshini et al. used linear discriminant analysis and support vector machine to distinguish thyroid nodules and compared the results of two methods [11]. Sathyapriya and Anitha selected the optimal features using Dynamic Mutation based Glowworm Swarm Optimization (DMGSO) algorithm and used the Long Short-Term Memory (LSTM) scheme to classify the thyroid nodules in 2020 [12]. In 2020, Ma et al. used five common machine learning methods to distinguish thyroid nodule; meanwhile, they also considered the impact of four different distance measures on the performance of the related models [13]. In 2020, Miao et al. used logistic regression to analyze the variables that significantly affected malignant nodules on the data of 811 patients with a total of 1290 pathologically confirmed nodules (506 benign and 784 malignant). The sensitivity and specificity of ultrasound thyroid imaging reporting and data system classification results for benign and malignant tumors were calculated [14]. In 2020, Ataide et al. aimed to reduce subjectivity in the current diagnostic process by using geometric and morphological (G-M) features that represent the visual characteristics of thyroid nodules to provide physicians with decision support [15].

Numerous CAD deep learning-based systems have been studied for automated thyroid detection in recent years. In 2017, Chi et al. used deep convolutional neural network to extract features from thyroid ultrasound images and sent the extracted features of the thyroid ultrasound images to a cost-sensitive random forest classifier to classify the images [16]. In 2019, Ouyang et al. compared the classification performance of five nonlinear and three linear machine learning algorithms for the evaluation of thyroid nodules. They obtained that nonlinear machine learning algorithms demonstrated similar AUCs compared with linear algorithms [17]. In 2019, Zhang et al. developed a thyroid nodules diagnostic model and performed better than radiologist diagnosis based on conventional US only and based on both conventional US and real-time elastography [18]. In 2020, Wang et al. compared the performance of radiomics and deep learning based methods for the classification of thyroid nodules from ultrasound images and found that the deep learning based method can achieve a better performance than using radiomics [19]. In 2020, Song et al. proposed a hybrid multibranch convolutional neural network based on feature cropping method for feature extraction and classification of thyroid nodule ultrasound images [20]. In 2020, Liang et al. developed a multiorgan CAD system based on convolutional neural networks for classifying both thyroid and breast nodules and investigated the impact of this system on the diagnostic efficiency of different preprocessing approaches. The authors proposed four models based on convolutional neural networks and found that the convolutional neural networks model using segmented images for classification achieved the best result [21]. In 2020, Zhang et al. proposed a novel method using two combined classification modules to separate and classify thyroid nodules images and experimental results show that the method leads to excellent performance [22]. In 2020, Xie et al. designed a deep neural network to classify whether a thyroid nodule is benign or malignant and proposed a structure which combines local binary pattern with deep learning [23]. In 2020, Yuan illustrated a method which involves the combination of the deep features with the conventional features together to form a hybrid feature space. They compared the ResNet18, a residual convolutional neural network with 18 layers, with the Res-GAN, a residual generative adversarial network [24]. In 2021, Liu et al. developed an information fusion-based joint convolutional neural network for the differential diagnosis of malignant and benign thyroid nodules. The information fusion-based joint convolutional neural network contains two branched convolutional neural networks for deep feature extraction [25]. In 2021, Vadhiraj et al. compared the support vector machine and artificial neural network classification algorithms based on their accuracy score, sensitivity, and specificity in the data of 99 thyroid nodule cases (33 benign and 66 malignant) [26]. In 2021, Li et al. designed an end-to-end thyroid nodule automatic recognition and classification system based on convolutional neural networks for CT images. The classification output accuracy reaches 0.8592 in the test set [27]. In 2022, Luong et al. used the linear, nonlinear, and nonlinear-ensemble machine learning methods to predict malignancy of indeterminate thyroid nodules and produced an accuracy of 0.791 [28]. Nair and Pande gave the analysis of different machine learning techniques used in the detection and prediction of thyroid disease in 2022 [29]. More information on ultrasonic image classification of thyroid nodules using deep learning method can be found in literature [30]. These results based on ultrasound images have good inspiration for our research.

The medical diagnosis system based on deep learning heavily depends on the amount of medical images. Sufficient data can ensure the performance of deep learning algorithm. However, thyroid nodule images data is very rare and expensive in the field of medical image processing. The lack of data has become a challenging problem in the classification of thyroid nodules using deep learning. In order to solve this problem, a deep neural networks classification model based on image enhancement technology is proposed in this paper. The introduction of image enhancement technology enables us to make full use of the limited image data information. The accuracy, precision, recall, and F1-score of the model would be important determinants of the clinical utility of the computer-aided diagnosis system. This paper uses the above four metrics as indicators to measure the performance of the classification models. The experimental results show that our proposed method achieves better performance compared with the existing thyroid nodule diagnosis systems in terms of accuracy, precision, recall, and F1-score.

## 2. Materials and Methods

Based on the previous literature and the opinions of doctors and experts, an accurate thyroid nodule detection and classification model is proposed based on deep neural networks and image enhancement.

### 2.1. Dataset

**Figure 1:**
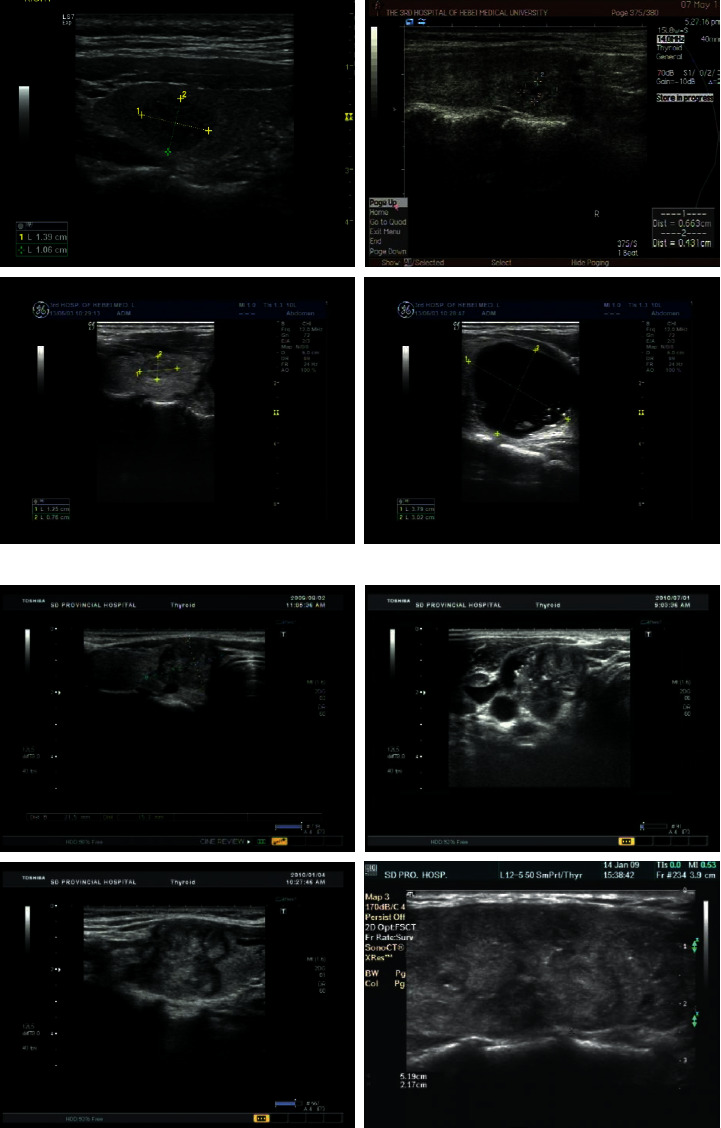
Examples of the original ultrasound image with thyroid nodules. (a) The thyroid nodules of the upper four images are benign. (b) The thyroid nodules of the lower four images are malignant.

A digital database of thyroid ultrasound images is established. These thyroid ultrasound images were collected at the third hospital of Hebei Medical University in China. The dataset images were divided into two categories: benign and malignant. The classification of thyroid nodules is the result of diagnosis according to the shape, nodule composition, shape, margin, calcifications, and TI-RADS score of thyroid nodules. A total of 508 patient ultrasound examinations were present in the dataset. Among these ultrasound images, there were 415 with nodules classified as benign and 93 with nodules classified as malignant. The specific original ultrasonic images data are shown in Figure 1.

### 2.2. Image Analysis and Preprocessing

Ultrasound images are collected from different doctors and equipment; image preprocessing is very necessary. These pieces of equipment include Philips IU22, Philips HD15, and Siemens Acuson SEQUOIA 512; the size of the images also ranges from 600 × 450 pixels to 768 × 576 pixels. All images had to be resized and changed to the same distance scale. In order to make better use of the images for the diagnosis of thyroid nodules, we preprocessed the ultrasound images in three steps.

**Figure 2:**
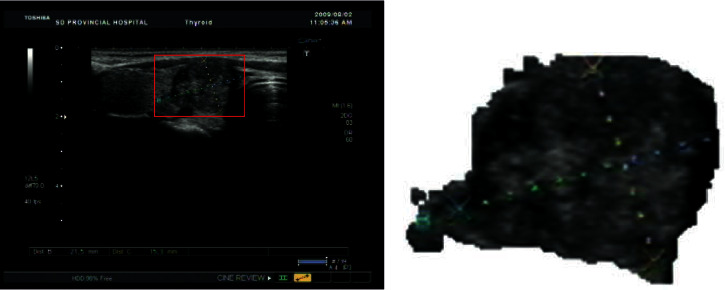
Image segmentation. (a) Original image. (b) Segmented and extracted image.

The first step of our image preprocessing is image segmentation, which is a process of segregating an image into numerous segments. Segmentation is required to change the representation of an image for easy analysis without altering meaningful information. Image segmentation is also an important step to extract redundant information from ultrasonic images.  Figure 2 exhibits the segmentation of an image.

The second step is to resize the images. It is resized to have the same distance scale which is the physical space represented by each pixel in the image. This step is used to improve the quality of the image by removing the undesired distortion from the image. The resizing of the image is also an important guarantee for the unity of the image data. In this study, the size of the image is uniformly adjusted to 200 × 200 pixels.

**Figure 3:**
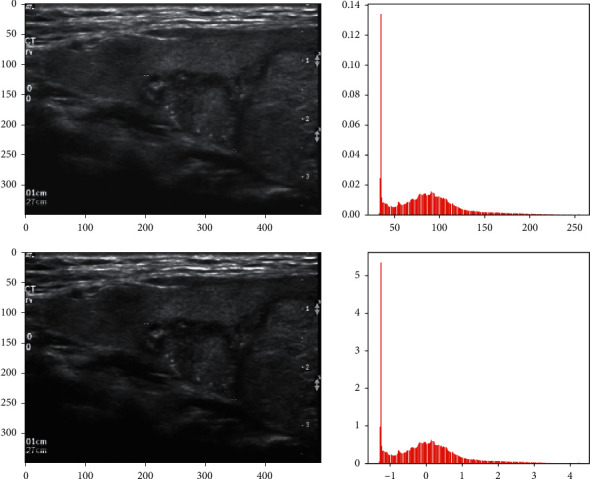
Image standardization.

Image standardization is the third step, also the final step of preprocessing. Image standardization is the process of centralizing the data by removing the mean value. According to the convex optimization theory and the data probability distribution, data centralization conforms to the law of data distribution, which makes it easier to obtain the generalization effect after training. Data standardization is one of the common methods of data preprocessing.  Figure 3 shows the changes of the image before and after image standardization.

### 2.3. Image Enhancement

Image enhancement algorithms are often used to adjust the brightness, contrast, saturation, and hue of the image, improve the definition of the image, and reduce the image noise. The purpose of image enhancement is to obtain the useful information of the image. Image contrast enhancement is the most important in image enhancement. There are two methods of image contrast enhancement: direct contrast enhancement method and indirect contrast enhancement method. Histogram equalization is a common indirect contrast enhancement method.

**Figure 4:**
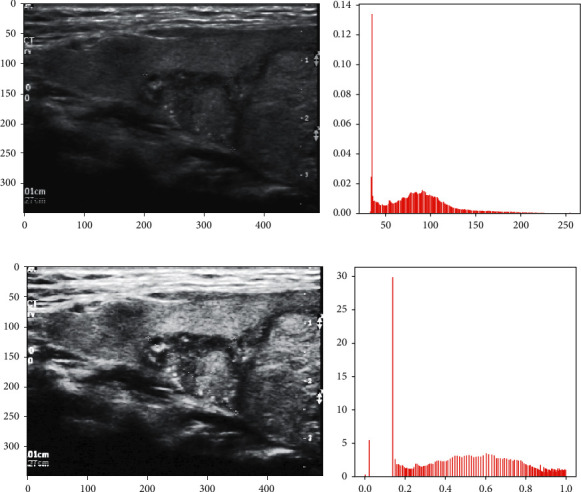
Histogram equalization. (a) The original image. (b) The histogram of the original image. (c) The histogram equalized image. (d) The histogram of the histogram equalized image.

The histogram of an image represents the probability density function (PDF) value of the pixel values in the image over the entire grayscale range. If most of the pixels are concentrated in the low gray area, the image will appear dark, but if they are concentrated in the high gray area, it will appear bright. Histogram equalization is to adjust the grayscale distribution of the image to make the distribution on the grayscale of 0∼255 more balanced. Histogram equalization uses the cumulative function to adjust the gray value to elevate the contrast of the image, thus improving the visual effect of the image.  Figure 4 shows the changes of the image and the histogram before and after histogram equalization.

### 2.4. Deep Neural Networks (DNNs)

**Figure 5:**
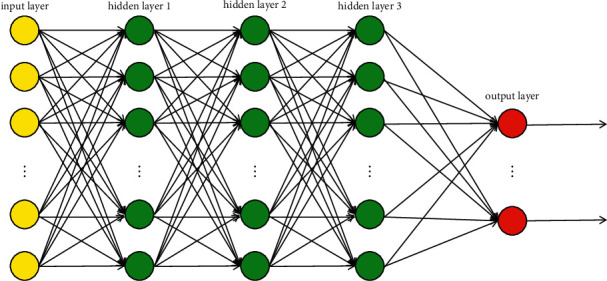
Deep neural networks.

Neural network is an extension based on the perceptron, and a DNN is a feed-forward, artificial neural network that has more than one layer of hidden units between its inputs and outputs. According to the location of different layers, the neural network layers of DNN can be divided into three categories: input layer, hidden layer, and output layer, as shown in Figure 5. The first layer is the input layer, the last layer is the output layer, and the middle layer is the hidden layer. Layers are fully connected; that is, any neural unit in layer *n* must be connected with any neural unit in layer *n* + *1*. Each hidden unit *j* typically uses the logistic function to map its total input from the layer below, *x*_*i*_, to the scalar state, *y*_*j*_, that it sends to the layer above. The logistic function is given by

(1) $$\begin{array}{c} y_{i}=\frac{1}{1+e^{-x_{i}}}, \end{array}$$

where *x*_*i*_=*b*_*j*_+∑_*i*_*y*_*i*_*w*_*ij*_, *b*_*j*_ is the bias of unit *j*, *i* is an index over units in the layer below, and *w*_*ij*_ is the weight on a connection to unit *j* from unit *i* in the layer in the following. Of course, there are other activation functions here. The logistic function is the most commonly used as the activation function.

DNN can be trained by back-propagating derivatives of the cost function that measures the discrepancy between the target outputs and the actual outputs produced for each training sample. For the large training sets, it is more efficient to use the strategy of “minibatch” for training deep neural networks model. That is to say, we compute the derivatives on a small, random “minibatch” rather than the whole training set, before updating the weights in proportion to the gradient.

### 2.5. Classification of Thyroid Nodules

**Figure 6:**
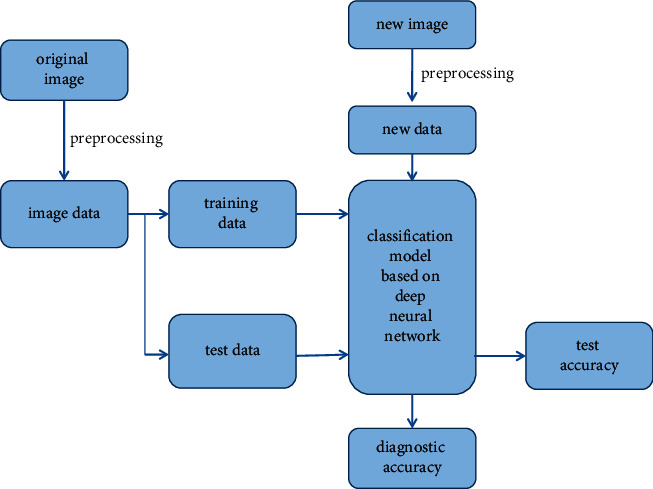
Framework of thyroid nodule classification based on deep neural networks.

Image classification task of thyroid nodules includes two parts: training and testing. After preprocessing the image data, we get the data that can be used directly. In order to make better use of image information, we carry out the image enhancement. Then, 80% of the data is used as training data and the other 20% as test data. DNN is chosen for classification. The classification accuracy, precision, recall, and F1-score are used as the standard to evaluate the classification model. At the same time, we also compare the classification results with the general machine learning classification algorithm, for example, K-nearest neighbors, decision tree, naive Bayesian model, support vector machine, logistic regression, and reinforcement learning. The process of thyroid nodule processing and classification based on deep neural networks is shown in Figure 6.

### 2.6. Performance Metric

In machine learning, it is very important to evaluate the model through performance measurement. In order to evaluate our proposed classification model based on deep neural networks, we compare the performance with those of the general machine learning classification algorithms. At the same time, we also consider the effects of different network structures, the number of iterations, and network activation functions on classification performance. In this paper, our focus is the accuracy of the model in building computer-aided diagnostics that could classify thyroid nodules as benign or malignant. However, it is also essential to check the performance and efficiency of the model. In order to evaluate the performance of the model more comprehensively, we consider the four following metrics: accuracy, precision, recall, and F1-score.

(1) Accuracy:
  (2) $$\begin{array}{c} \text{accuracy}=\frac{\text{TP}+\text{TN}}{\text{TP}+\text{FP}+\text{TN}+\text{FN}}. \end{array}$$
(2) Precision:
  (3) $$\begin{array}{c} \text{precision}=\frac{\text{TP}}{\text{TP}+\text{FP}}. \end{array}$$
(3) Recall:
  (4) $$\begin{array}{c} \text{recall}=\frac{\text{TP}}{\text{TP}+\text{FN}}. \end{array}$$
(4) F1-score:

(5) $$\begin{array}{c} \text{F}1-\text{score}=2*\frac{\left(\text{precision}*\text{recall}\right)}{\text{precision}+\text{recall}}, \end{array}$$

where TP is True Positive, FP is False Positive, TN is True negative, and FN is False Negative.

Accuracy is one of the most important indexes in the performance metrics. Accuracy refers to how well the model can classify thyroid nodules correctly. Accuracy is the proportion of correctly predicted cases in the total cases. Precision is the proportion of positive cases determined by the classifier. Recall is the proportion of predicted positive cases in the total positive cases. F1-score is the harmonic average of precision and recall. Precision and recall have the same weight in F1-score.

## 3. Experimental Results and Discussion

### 3.1. Accuracy-Based Deep Neural Networks

In this study, we construct a thyroid nodule classification model based on deep neural networks. Classification of thyroid nodules using deep neural networks at the best setting reaches accuracy of 0.901961. Precision and F1-score also reach 0.894737 and 0.944444; in particular, recall reaches 1. The best network has four layers in our experiment, the first layer is the input layer, the last layer is the output layer, and the two middle layers are the hidden layers; that is, there are two hidden layers. We scale the image with 200 × 200 pixels to an image with 50 × 50 pixels and transform an image with 50 × 50 pixels into a 2500-dimensional vector for deep neural networks. Therefore, the number of nodes of the input layer is 2500. The node numbers of the two hidden layers are 40 and 2. Because it is a binary classification problem, the output layer has only one node. The activation function of the network node is the logistic function. The maximum number of iterations during model training is set to 500. Due to the difference between image acquisition channels and doctors, the image quality is relatively low. In particular, the recognition of some images is very difficult, and even manual recognition is difficult. In this case, the accuracy of deep neural networks classification model is satisfactory.

### 3.2. Influence of Different Neural Networks

In this section, we consider the influence of different neural networks. We first consider the influence of network structure on classification performance. For convenience, 2500-200-100-1 network denotes a four-layer network. 2500 is the number of network nodes in the first layer, that is, the number of network nodes in the input layer. 200 and 100 are the numbers of nodes in the second layer and the third layer; these two layers are hidden layers. 1 is the number of the output layers (the last layer).  Table 1 gives the performance comparison of DNN with different network structures. The network has more layers and network nodes, which means that the network will need more training time. From our experimental results, it is not true that the more complex the network structure, the better the performance of the model. It requires us to make more attempts to find the optimal network structure. We mainly consider three-layer, four-layer, and five-layer network structures. Six network structures are studied for each type of networks. We can find that the optimal network structure is four layers, 2500-40-2-1. Although the accuracy of a three-layer network (2500-100-1) also reaches 0.901961, the recall rate and F1-score are lower than those of the 2500-40-2-1 network. The recall and F1-score of the 2500-40-2-1 network are the highest in our experimental network structures; in particular, the recall reaches 1.

Next we discuss the influence of different activation functions. In order to consider only the influence of activation function, we set the same network structure, the number of iterations, and the learning rate. The network is the four-layer network (2500-40-2-1). The number of iterations and learning rate are set to 500 and 0.001, respectively. Logistic, tanh, and identity functions are considered. Logistic function is also sigmoid function. The formula of logistic function is seen in (1). The formulas of tanh and identity functions are as follows:

(6) $$\begin{array}{c} \text{tanh}=\frac{1-e^{-2x}}{1+e^{-2x}}=\frac{e^{x}-e^{-x}}{e^{x}+e^{-x}},\\ \text{identity}\left(x\right)=x. \end{array}$$

The performance comparison of DNN with different activation functions is shown in Table 2. It is obvious that logistic activation function has the best performance. The four performance metrics of logistic function are all higher than tanh and identity activation functions. The performance of tanh activation function is slightly higher than that of identity function.

Finally, we consider the influence of the number of iterations during training. In the process of neural network model training, we need to iterate many times in order to achieve better training effect. The selection of iteration times is very important for model training. Too many iterations easily leads to a large amount of redundant training time and overfitting; on the contrary, too few iterations leads to poor training effect. The performance comparison of DNN with different maximum number of iterations is given in Table 3. It is easy to find that the overall classification performance of the model improves with the increase of the number of iterations. Although in the process of improvement one or two indicators will decline, when the number of iterations reaches 500, the performance of the model is the best. The classification accuracy reaches 0.901961, and the precision, recall, and F1-score reach 0.894737, 1, and 0.944444, respectively. After 500 iterations, even if the number of iterations increases, the classification performance remains unchanged. On the contrary, it will cause a violent increase in training time.

### 3.3. Comparison with Other Methods

In this section, we compare the performance of deep neural networks with those of other common machine learning classification methods.  Table 4 lists the comparison of deep neural networks and other six methods: K-nearest neighbors, decision tree, naive Bayesian model, support vector machine, logistic regression, and reinforcement learning. In the six methods, we choose the best results of each method for comparison. In K-nearest neighbors, we choose *K* = 3, which is also the best classification performance of K-nearest neighbors. In naive Bayesian method, we consider the Gaussian model, which is the most popular naive Bayesian model. Linear kernel function is used in support vector machine. We consider two reinforcement learning methods: hard voting and soft voting.

The performance of DNN is obviously the best among all methods. The accuracy of DNN reaches 0.901961. The precision, recall, and F1-score reach 0.894737, 1, and 0.944444, respectively. All four performance metrics of deep neural networks are the highest in our experimental methods. In other six methods, reinforcement learning (soft voting), K-nearest neighbors, and support vector machine have better classification performance. The recall of soft voting reaches 1; however, the accuracy, precision, and F1-score performance metrics are significantly lower than those of deep neural networks. The accuracy of K-nearest neighbors is closest to that of deep neural networks; the accuracy is 0.852941. The naive Bayesian model has the worst classification performance.

### 3.4. Influence of Image Enhancement

The image enhancement method used in this paper is histogram equalization. Histogram equalization is to adjust the grayscale distribution of the image to make the distribution on the gray more balanced. Equalization helps to extend the image histogram. After equalization, the gray level range of the image is wider and the contrast of the image is effectively enhanced.  Table 5 shows the performance comparison of deep neural networks with and without image enhancement. We can easily find the influence of image enhancement on classification performance. After image enhancement, all four performance metrics of deep neural networks have been improved greatly. The accuracy increases from 0.715686 to 0.901961. Other classification algorithms have similar results. The classification performance of the model is improved with different degree after image enhancement.

## 4. Conclusions

In this paper, we proposed a thyroid nodule diagnosis method based on deep neural networks and image enhancement. Experimental results show that our method has superior performance as compared to other common classification algorithms. Because the experimental data are collected by different doctors from the hospital, some images are very blurred. In order to maintain the integrity of the data, we did not abandon the blurred images. Therefore, the classification performance of the classification model is relatively low in our experimental data. The classification accuracy still reaches 0.901961; in particular, the recall reaches 1. At the same time, we considered the influence of network structure on the classification results. We found that it is very difficult to find the optimal network structure. The determination of the optimal network structure is related not only to the network itself but also to the data to be processed. At present, we can only determine the optimal network structure through the experimental results. The experimental results show that the optimal network structure is 2500-40-2-1 in this paper. To determine the optimal network structure from the perspective of theoretical analysis is our next work.

We also considered the influence of activation function and the number of training iterations. The optimal activation function is logistic function and the best number of iterations is 500. In the last part of this paper, we considered the influence of image enhancement on classification performance. The accuracy before image enhancement is 0.715686 and the accuracy after image enhancement reaches 0.901961. The precision, recall, and F1-score have been improved after image enhancement.

## Figures and Tables

**Table 1:** Performance comparison of DNN with different network structures.

Number of network layers	Network structure	Accuracy	Precision	Recall	F1-score
Three layers	2500-50-1	0.843137	0.863158	0.964706	0.911111
	2500-60-1	0.882353	0.892473	0.976471	0.932584
	2500-80-1	0.892157	0.893617	0.988235	0.938547
	2500-100-1	0.901961	0.912088	0.976471	0.943182
	2500-200-1	0.892157	0.893617	0.988235	0.938547
	2500-300-1	0.882353	0.901099	0.964706	0.931818

Four layers	2500-10-2-1	0.823529	0.874045	0.917647	0.896552
	2500-20-2-1	0.833334	0.869565	0.941176	0.903955
	2500-30-2-1	0.862745	0.881720	0.964706	0.921348
	**2500-40-2-1**	**0.901961**	**0.894737**	**1**	**0.944444**
	2500-60-2-1	0.823529	0.876404	0.917647	0.896552
	2500-40-10-1	0.872549	0.8912304	0.964706	0.926554
	2500-40-20-1	0.852941	0.880435	0.952941	0.915254

Five layers	2500-40-5-2-1	0.843137	0.8555670	0.976471	0.912088
	2500-100-5-2-1	0.882353	0.901099	0.964706	0.931818
	2500-200-10-2-1	0.872549	0.882979	0.976471	0.927374
	2500-200-20-2-1	0.803921	0.857143	0.917647	0.886364
	2500-200-50-2-1	0.843137	0.896552	0.917647	0.906976
	2500-500-50-2-1	0.852941	0.880435	0.952941	0.915254

**Table 2:** Performance comparison of DNN with different activation functions.

Activation function	Accuracy	Precision	Recall	F1-score
Logistic	**0.901961**	**0.894737**	**1**	**0.944444**
Tanh	0.882353	0.884211	0.988235	0.933333
Identity	0.858586	0.861702	0.987805	0.920455

**Table 3:** Performance comparison of DNN with different number of iterations.

Maximum number of iterations	Accuracy	Precision	Recall	F1-score
80	0.862745	0.89010	0.952941	0.920455
100	0.882353	0.892473	0.976471	0.932584
150	0.843137	0.879121	0.941176	0.909091
200	0.892156	0.893617	0.988235	0.938547
**500**	**0.901961**	**0.894737**	**1**	**0.944444**
1000	0.901961	0.894737	1	0.944444
2000	0.901961	0.894737	1	0.944444

**Table 4:** Performance comparison of different classification methods.

Method	K-nearest neighbors	Decision tree	Naive Bayesian model	Support vector machine	Logistic regression	Reinforcement learning		Deep neural networks
						Hard voting	Soft voting	
Accuracy	0.852941	0.794118	0.411765	0.823529	0.823529	0.823529	0.843137	**0.901961**
Precision	0.857143	0.847826	0.329412	0.868132	0.860215	0.860215	0.841584	**0.894737**
Recall	0.988235	0.917647	0.903226	0.929412	0.941176	0.941176	1	**1**
F1-score	0.918033	0.881356	0.482759	0.897727	0.898876	0.898876	0.913978	**0.944444**

**Table 5:** Performance comparison of deep neural networks with and without image enhancement.

Image enhancement	Accuracy	Precision	Recall	F1-score
Without	0.715686	0.878378	0.764706	0.817610
With	**0.901961**	**0.894737**	**1**	**0.944444**

## Data Availability

The datasets generated and/or analyzed during the current study are available from the corresponding author upon reasonable request.
